# 

**Published:** 1871-10

**Authors:** 


### TO OUR SUBSCRIBERS.

It would be too troublesome and expensive to forward bills of subscription in every number of The Companion—we therefore, earnestly ask all of our subscribers who have not paid their subscription, the very small sum of two dollars, to do so now, and you will receive your bill, receipted, in the next number. Friends, don't forget to promptly discharge this part of your duty towards enabling our publisher to furnish The Companion monthly to you and your Professional brethren.

